# SPINK1 Mutation in Idiopathic Chronic Pancreatitis: How Pertinent Is It in Coastal Eastern India?

**DOI:** 10.7759/cureus.14427

**Published:** 2021-04-12

**Authors:** Subhra S Jena, Girish K Pati, Kanishka Uthansingh, G Vybhav Venkatesh, Pradeep Mallick, Manas Behera, Jimmy Narayan, Debakanta Mishra, Shobhit Agarwal, Manoj K Sahu

**Affiliations:** 1 Molecular Diagnostic and Research Center, Institute of Medical Sciences and SUM Hospital, Siksha 'O' Anusandhan Deemed to be University, Bhubaneswar, IND; 2 Department of Gastroenterology, Institute of Medical Sciences and SUM Hospital, Siksha 'O' Anusandhan Deemed to be University, Cuttack, IND; 3 Department of Gastroenterology, Institute of Medical Sciences and SUM Hospital, Siksha 'O' Anusandhan Deemed to be University, Bhubaneswar, IND

**Keywords:** chronic pancreatitis, genetic mutation, pain abdomen, spink1 gene

## Abstract

Background and aim

Idiopathic chronic pancreatitis (ICP) is said to be present when no identifiable etiology can be identified. Robust evidence suggested that the serine protease inhibitor nucleus Kazol type 1 (SPINK1) N34S mutation was frequently associated with ICP. As there is a paucity of data on genetic studies in ICP cases from the coastal eastern region of India, we performed this study with an aim to evaluate the SPINK1 genetic mutations and other associated clinical correlates in ICP cases.

Material and methods

Consecutive ICP cases attending the department of gastroenterology, Institute of Medical Sciences (IMS) and SUM Hospital, were enrolled and evaluated for the pertinent clinical history and undergone detailed biochemical and radiological evaluations. Two ml of venous blood in ethylenediaminetetraacetic acid (EDTA) vials were collected from each case and subjected to a polymerase chain reaction-restriction fragment length polymorphism (PCR-RFLP) test for genetic analysis.

Result

In this study, the mean age of the cases at the time of the first consultation with us and the age of the first clinical presentation were 34.52±6.44 and 28.73±5.52 years, respectively. Males outnumbered females (Male:Female - 2.12:1). Out of the total of 200 cases, 50% had no SPINK1 mutation, whereas 40% and 10% cases had SPINK1 N34S heterozygous and homozygous mutations, respectively. The mean age of clinical presentation, severe abdominal pain, exocrine and endocrine insufficiency, and parenchymal atrophy were significantly more common in mutants as compared to non-mutants (p-value <0.05).

Conclusion

In our region, 50% of ICP cases had the SPINK1 N34S mutation. The SPINK1 mutants had a relatively more severe variety of pancreatitis as compared to non-mutants.

## Introduction

Chronic pancreatitis (CP) is described as a chronic inflammatory complex disorder of the pancreas, resulting in irreversible morphological changes and manifests usually with recurrent or chronic abdominal pain initially and with exocrine and/or, endocrine insufficiencies in the long run [1-2]. Most patients with CP usually suffer from a relapsing and remitting type of abdominal pain initially and may suffer from maldigestion, diabetes mellitus, and pancreatic cancer later on if they were improperly managed [3]. Although a lot of putative factors for the development of CP were described in the literature, in nearly 10%-30% of the cases, no identifiable cause could be identified, and these cases were labeled as idiopathic chronic pancreatitis (ICP) [4]. A lot of research has been carried out on acute and CP as a whole, but very few studies have characteristically focused on ICP, a distinct form of CP, which remains a mystery to date. A previous report suggested that ICP was prevalent only in certain geographic regions of India [4]. In the preceding few years, many cases of ICP have been reported not only from different regions of the western world but also from most states of India too [5]. It was hypothesized that ICP is a complex disorder, which may occur due to varied interaction of genetic mutations and the environment [6-7]. Various genetic mutations in different putative genes such as the cationic trypsinogen (PRSS1) gene, serine protease inhibitor nucleus Kazol type 1 (SPINK1) gene, cystic fibrosis transmembrane conductance regular (CFTR) gene, and cathepsin B gene have been well-described in acute, recurrent, and chronic pancreatitis [8]. ICP is usually associated with a mutation in the SPINK1 gene, but its true prevalence in the community over different geographic regions was not well-studied to date due to the non-availability of molecular and genetic laboratories in the concerned regions. The SPINK1 gene is usually located on chromosome 5 and acts as the first line of defense against the development of pancreatitis, as it limits the premature activation of trypsinogen inside the zymogen granules of the pancreas by competitively blocking the active site of trypsin, by which the autodigestion of pancreatic parenchyma could be prevented [8-9]. In case of a SPINK1 mutation or alteration, pancreatitis may set in due to the unabated activity of trypsin [10]. Previous studies by Gomez-Lira M et al. and Shetty S et al. revealed that genetic mutation may be the most important causative factor of ICP [7,11]. Usually, the most common mutation in the SPINK1 gene occurs in exon 3 at codon 34, which results in a change of amino acid from asparagine to serine (N34S), by which trypsin inhibitory capacity gets compromised. Previous studies suggested that the SPINK1 N34S mutation was fairly common in ICP cases [6,12-13]. To date, as there is a paucity of genetic studies in ICP cases from this part of the coastal eastern region, we carried out this targeted genetic study with an aim to evaluate the prevalence of the SPINK1 N34S mutation in cases with ICP and to correlate it with different clinical and morphological presentations.

## Materials and methods

This study was a facility-based, cross-sectional, open-labeled, non-interventional cohort study that was carried out for a duration of 11 months in 2020.

In this study, consecutive ICP cases attending the department of gastroenterology, Institute of Medical Sciences (IMS) and SUM Hospital, Bhubaneswar, Odisha, were enrolled and evaluated in detail about pertinent clinical presentations and family history; and their genetic studies were carried out in the Molecular Diagnostic and Research Center, IMS and SUM Hospital. Written informed consent was obtained from all the cases, and the study protocol was approved by the institutional ethical committee. All the cases were subjected to pertinent biochemical tests and radiological evaluation by abdominal ultrasonography and contrast-enhanced computed tomography (CECT) study. Complications of CP such as diabetes mellitus (DM), steatorrhea, distal biliary stricture, and pseudocyst were diagnosed on the basis of standard clinical, biochemical, and radiological criteria. Chronic pancreatitis was said to be present when at least two separate episodes of pancreatic type of abdominal pain and typical radiological findings such as pancreatic parenchymal calcifications, parenchymal atrophy, intraductal stones, and ductal dilatations were noticed [3]. All these structural abnormalities may be variably present in ICP cases. Pertinent clinical history included etiology, type, and severity of pain, age of first clinical presentation, total disease duration, presence or absence of fatty diarrhea (steatorrhoea) and diabetes mellitus, etc. CP cases with underlying etiological factors, such as a history of significant alcohol drinking (daily intake ≥ of 80 gram of ethanol for at least two years), cholelithiasis, infection, trauma, medications, surgery, metabolic disorders, such as hypercalcemia (serum calcium ≥ 12 mg/dl) and hypertriglyceridemia (serum triglyceride ≥ 1000 mg/dl), and a family history of pancreatitis (presence of at least two affected first-degree relatives or three or more second-degree relatives in two or more generations) [3,14-15] were excluded from the study. Steattorhoea was diagnosed subjectively, whenever patients passed oily and/or sticky stool. Diabetes mellitus (DM) was diagnosed if fasting blood glucose (FBS) value was ≥126 mg/ dl and/ or two-hour plasma glucose (two-hour post-glucose blood sugar (PGBS)), or random blood glucose (RBS) value ≥200 mg/dl [16].

Procedures for mutational analysis

The genotyping procedure starts from the collection of the patient’s peripheral blood sample in EDTA-containing tubes, extraction of deoxyribonucleic acid (DNA), carrying out polymerase chain reaction (PCR) by using the targeted primer for SPINK1 and restriction fragment length polymorphism (RFLP) by using the BsrDI and PstI restriction enzymes. The mutational changes were assayed by analyzing the developed digested and undigested products in form of bands through the Biorad gel doc imaging system.

1. Sample collection: Approximately 2 ml of venous blood was collected from each of the participants for genetic analysis. The peripheral blood samples were collected in EDTA-containing tubes followed by storage in a refrigerator for further processing.

2. DNA extraction: Genomic DNA was extracted and purified from whole blood by using the salting-out method, which was further utilized for polymerase chain reaction (PCR) to study genetic polymorphism. The isolated DNA was converted to a concentration of 80-100 ng for further PCR analysis.

3. Polymerase chain reaction-restriction fragment length polymorphism (PCR-RFLP): The N34S mutation was assayed by the PCR-RFLP technique. Designed primers were used to amplify exon 3 on the basis of specific nucleotide sequences (GenBank, NM-003122). Proofreading activity may be carried out from the 3’ to 5’ location during the PCR test for the elimination of any PCR-related error if required. PCR was performed by using a genomic DNA template. The forward and reverse primer sequences were 5’-TTC TGT TTA ATT CCA TTT TTA GGC CAA ATG CTG CA-3’ and 5’-GGC TTA CAT ACA AGT GAC TTC T-3’, respectively. The primers were designed to introduce a PstI endonuclease restriction site in sequences having N34S mutation and a BsrDI endonuclease restriction site at wild-type sequences. Before doing PCR, we made the aliquot; the powder form of primer by taking nuclease-free water and stock primer. The isolated DNA was converted to a concentration of 80-100 ng for further steps. Subsequently, for further PCR procedure, we used the Dream Taq Green PCR master mix to assess the reaction. Denaturation was done by setting the heat temperature to 93°c-95°c; the reaction mixture was heated at 95°C for a brief period to denature the double-stranded target DNA into a single-stranded form, which can act as a template for DNA synthesis. The annealing temperature was set to 55°C-60°C; the same mixture subsequently quickly cooled to a defined temperature, which is used to permit the primer/s to bind target groups on both the strands of flanking targeted DNA. The extension temperature was set to 71°C-72°C; during this step, the temperature of the reaction mixture was again elevated to 72°C to allow the enzyme to synthesize the single-stranded template DNA. Finally, the extension temperature was set at 72°C. Initially, the stock primer was converted to a working primer according to the protocol sheet by Thermo Fisher Scientific Company (Waltham, Massachusetts) by adding 90 µl nucleus-free water with 10 µl stock primer (forward and reverse). After preparation of the master mix, it was mixed properly by the use of a vortex, and out of it, 9.5 µl and 12.5 µl were transferred to PCR tubes sequentially to assess the reaction. Then the PCR tubes were made bubble-free by tapping and rotated for proper mixing. Following completion of all the PCR steps, we assayed the amplified bands in 2% gel by running gel electrophoresis. The image developed in the gel was visualized under gel doc. All the PCR byproducts were subjected to digestion procedures by the use of restriction endonucleases PstI and BsrDI as per the protocol. Subsequently, it was thawed vigorously and incubated at 55°C for 1 hour. Then, the products were analyzed by agarose gel electrophoresis by the use of 3% (w/v) Nusieve 3:1 agarose. The genotypes were determined on the basis of the expected product size {A/A Genotype (Asn 34 Asn) - 320bp, G/G Genotype (Ser 34 Ser) - 286bp, A/G Genotype (Asn 34 Ser) - 320,286 and 34}.

4. Statistical analysis: All the results were expressed as mean ± standard deviation (SD) or frequency (in percent). The quantitative and categorical variables were compared by using the student’s t-test and chi-square test, respectively. All the analyses were performed by using the Statistical Package for the Social Sciences (SPSS) 22 software (IBM Corp., Armonk, NY). A p-value of <0.05 was considered statistically significant.

## Results

In this study, 200 consecutive ICP cases were included. Males outnumbered females (Males:Females - 2.12:1). The mean age of presentation at baseline and first clinical presentation were 34.52±6.44 and 28.73±5.52 years, respectively. The mean disease duration was 5.94±3.36 years. The different characteristics of ICP cases and their comparative analysis are described in Tables 1-2, respectively. Most (95.5%) of the cases presented with the pancreatic type of abdominal pain. Exocrine insufficiency in the form of steatorrhoea was complained of by 20.5% cases, whereas 19% of cases suffered from endocrine insufficiency in the form of type 2 DM. The clinical presentations of ICP cases and their comparative analyses are presented in Tables 3-4, respectively. The presence of a distal biliary stricture and pancreatic pseudocyst was noticed in 11.5% and 14% cases, respectively. Most (70.5%) cases had a pancreatic structural abnormality in form of parenchymal calcifications; whereas 34.5% cases had parenchymal atrophy. Sixty-five point five percent (65.5%) and 60.5% cases had a dilated pancreatic duct and intraductal calculi, respectively. The structural changes of ICP cases and their comparative analyses were illustrated in Tables 5-6 respectively. In our study subjects, 50% of cases had no mutational changes or wild-type sequences of the SPINK1 gene, whereas 40% and 10% cases had SPINK1 heterozygous and homozygous mutations, respectively. A comparative analysis of all the characteristics in between SPINK1 mutated and un-mutated cases Is described in Table 7.

**Table 1:** Different characteristics of ICP cases ICP: idiopathic chronic pancreatitis

Characteristics	Total cases (n – 200)	Cases with homozygous mutation (n – 20)	Cases with heterozygous mutation (n – 80)	Un-mutated cases (n – 100)
Male:Female ratio	2.12:1	4:1	1.85:1	2.12:1
Mean age of presentation at baseline in years	34.52±6.44	26.7±5.32	32.49±5.82	37.71±5
Mean age of first clinical presentation in years	28.73±5.52	21.75±2.24	25.94±3.51	32.36±4.59
Mean disease duration in years	5.94±3.36	4.95±3.35	6.54±3.19	5.65±3.45

**Table 2:** Comparative analysis of different characteristics of ICP cases ICP: idiopathic chronic pancreatitis

Characteristics	p-value in between homozygote and heterozygote	p-value in between homozygote and un-mutated cases	p-value in between heterozygote and un-mutated cases
Male:female ratio	0.19	0.25	0.67
Mean age of presentation at baseline in years	0.0001	0.0001	0.0001
Mean age of first clinical presentation in years	0.0001	0.0001	0.0001
Mean disease duration in years	0.051	0.4	0.07

**Table 3:** Clinical presentations of ICP cases n - Number ICP: idiopathic chronic pancreatitis

Clinical presentations	Total cases (%) (n – 200)	Cases with homozygous mutation (%) (n – 20)	Cases with heterozygous mutation (%) (n – 80)	Un-mutated cases (%) (n – 100)
Abdominal Pain	95	100	100	94
Non-Severe Abdominal Pain	64	25	52.55	84
Severe Abdominal Pain	31	75	47.5	10
Passage of Fatty Stool	20.5	45	25	12
Presence of Diabetes Mellitus	19	35	25	11

**Table 4:** Comparative analysis of clinical presentations of ICP cases ICP: idiopathic chronic pancreatitis

Clinical presentations	p-value in between homozygote and heterozygote	p-value in between homozygote and un-mutated cases	p-value in between heterozygote and un-mutated cases
Non Severe Abdominal Pain	0.03	<0.0001	<0.0001
Severe Abdominal Pain	0.02	<0.0001	<0.0001
Passage of Fatty Stool	0.07	0.0004	0.02
Presence of Diabetes Mellitus	0.36	0.0006	0.01

**Table 5:** Structural changes in ICP cases n - Number ICP: idiopathic chronic pancreatitis

Structural changes	Total cases (%) (n – 200)	Cases with homozygous mutation (%) (n – 20)	Cases with heterozygous mutation (%) (n – 80)	Un-mutated cases (%) (n – 100)
Parenchymal Calcification	70.5	100	53.75	78
Parenchymal Atrophy	34.5	25	50	24
Intraductal Calculi	60.5	90	60	55
Dilated Pancreatic Duct	65.5	90	63.75	62
Distal Biliary Stricture	11.5	25	13.75	7
Pseudocyst	14	20	18.75	9

**Table 6:** Comparative analysis of structural changes of ICP cases ICP: idiopathic chronic pancreatitis

Structural changes	p-value in between homozygote and heterozygote	p-value in between homozygote and un-mutated cases	p-value in between heterozygote and un-mutated cases
Parenchymal Calcification	0.0001	0.02	0.0004
Parenchymal Atrophy	0.04	0.92	0.003
Intraductal Calculi	0.01	0.003	0.5
Dilated Pancreatic Duct	0.02	0.01	0.89
Distal Biliary Stricture	0.18	0.01	0.17
Pseudocyst	0.83	0.14	0.07

**Table 7:** Comparative analysis between SPINK1 mutated and un-mutated cases SPINK1: serine protease inhibitor nucleus Kazol type 1

Characteristics	SPINK1 mutated cases	SPINK1 un-mutated cases	p-value
Male:Female Ratio	2:1	2.12:1	0.88
Mean Age of Presentation at Baseline in Years	31.33±6.16	37.71±5	0.0001
Mean Age of First Clinical Presentation in Years	25.1±3.69	32.36±4.59	0.0001
Mean Disease Duration in years	6.22±3.27	5.65±3.45	0.23
Abdominal Pain (%)	100	90	0.0012
Severe Abdominal Pain (%)	52	10	<0.0001
Passage of Fatty Stool (%)	29	12	0.0029
Presence of Diabetes Mellitus (%)	27	11	0.0039
Parenchymal Calcification (%)	64	78	0.02
Parenchymal Atrophy (%)	49	24	0.0002
Intraductal Calculi (%)	66	55	0.11
Dilated Pancreatic Duct (%)	69	62	0.29
Distal Biliary Stricture (%)	16	07	0.04
Pseudocyst (%)	19	09	0.04

## Discussion

Males outnumbered females in our study, as similarly reported by other Indian studies by Shetty S et al. [11] and Balakrishnan V et al. [17]. The mean age of presentation at baseline was 34.52±6.44 years, which was consistent with study findings by Shetty S et al. [11], Balakrishnan V et al. [18], and Garg PK et al. [19]. The mean duration of the disease in our cases was 5.94±3.36 years, whereas it was 31 months, 48 months, and 27 months as reported by Shetty S et al. [11], Garg PK et al. [20], and Midha S et al. [21]. Abdominal pain was the most common clinical presentation in our study, which was in agreement with the findings by Balakrishnan V et al. [18], Midha S et al. [21], and Layer et al. [22]. History suggestive of steatorrhoea was present in 20.5% of our study population, whereas its prevalence was 5%, 15%, and 5% in studies by Midha et al. [21], Shetty S et al. [11], and Garg PK et al. [20], respectively, which indicated that steatorrhoea was relatively more common in ICP cases from our region as compared to other regions. We noticed that 19% of our study population had type 2 DM, whereas its prevalence was 33%, 26%, and 27% as observed by Shetty S et al. [11], Garg PK et al. [20], and Midha S et al. [21], respectively, which suggested that ICP cases from our region had a lower prevalence of DM in comparison to cases from other geographic regions. We observed that 70.5%, 65.5%, and 60.5% of cases from our region had pancreatic parenchymal calcification, dilated pancreatic duct (PD), and intraductal calculi respectively; on the contrary, Shetty S et al. noticed that 87.87%, 75%, and 21% of cases had these type of structural abnormalities respectively in his study group [11]. We observed that, amongst all the ICP cases from our region, 50% cases had no genetic mutation in the SPINK1 gene or had the wild variety of the SPINK1 gene, whereas the rest 50% cases had the SPINK1 N34S mutation (40% were heterozygous, and 10% were homozygous). Shetty S et al. [11], Garg PK et al. [20], Bhatia et al. [23], and Chandak GR et al. [24] showed that 36.36%, 42%, 40%, and 32.5% of their cases had the SPINK1 mutation, respectively, which supported our study findings. We noticed that out of all the 100 SPINK1 N34S mutated cases, 20% of cases were homozygote, which was consistent with the result reported by Chandak GR et al. [24], who found that 18.42% of SPINK1 N34S mutated cases were homozygotes. The prevalence of the SPINK1 mutation in ICP cases from our region was 50%, which was relatively higher compared to findings of other published reports such as 6.4% by Chen JM et al. [25], 18.0% by Threadgold J et al. [26], 21.0% by Drenth JP et al. [27], and 40.4% by Pfutzer et al. [6]. We also noticed that 64% of cases with SPINK1 mutation had parenchymal calcification, which was much less in comparison to findings by Shetty S et al. [11], who found parenchymal calcification in all the SPINK1 mutated cases in his study group. We also observed that 49% of cases with SPINK1 mutation had pancreatic parenchymal atrophy, which was much less compared to the study result by Shetty S et al. [11], who reported 75% of cases with the SPINK1 mutation had parenchymal atrophy. In our study, although the mean age of the first clinical presentation and presentation at baseline in mutated cases were significantly earlier as compared to non-mutated cases, the mean disease duration was not significantly different (p>0.05) in between them. Mutated cases had a higher occurrence of severe abdominal pain, steatorrhoea, and type 2 DM as compared to un-mutated cases. We also observed that mutated cases had a significantly higher prevalence of pancreatic parenchymal atrophy, distal biliary stricture, and pancreatic pseudocyst in comparison to their un-mutated counterparts, although the prevalence of intraductal calculi and dilated pancreatic duct were not significantly different in between them. We also observed that homozygote cases had a relatively earlier mean age of first clinical presentation and presentation at baseline when compared to heterozygote cases. Although homozygote cases had a higher occurrence of severe abdominal pain as compared to heterozygote, they had no significant difference in the prevalence of fatty diarrhea and type 2 DM in between them. We, too, found that homozygote had a significantly higher prevalence of parenchymal calcification, ductal dilatation, and intraductal calculi as compared to heterozygote, although the prevalence of pancreatic pseudocyst and distal biliary stricture was not different between them. Surprisingly we found that un-mutated cases had a significantly higher prevalence of pancreatic parenchymal calcification as compared to mutated cases and heterozygote had a higher prevalence of pancreatic parenchymal atrophy as compared to homozygote, which suggests that, possibly, SPINK1 mutation is not a predominant disease inducer as earlier presumed; rather, it may be a disease modifier too and its effect may be modified by the environment and other associated mutated genes if present. Truninger K et al. [28] suggested that a stronger genetic abnormality may be an etiologic event for early-onset ICP as compared to late-onset ICP, but the study by Chandak GR et al. [24] did not support his finding. Chandak GR et al. [24] found no significant phenotypical differences between SPINK1 mutated and un-mutated cases and between SPINK1 N34S heterozygote and homozygote, although he observed that homozygote had a higher prevalence of type 2 DM as compared to heterozygote. Threadgold J et al. [26] reported that the SPINK1 N34S mutation was neither associated with earlier disease onset nor a severe type of chronic pancreatitis. He also noted that there were no significant differences between N34S mutated and un-mutated cases in terms of age of onset of pancreatic exocrine and endocrine insufficiency, frequency and duration of attacks, number of hospitalizations, age of first surgery, and surgical repair for complicated pancreatitis [26]. In contrast to the above study findings, the study by Muller N et al. reported that SPINK1 mutated cases had a higher prevalence of acute pancreatic episodes, more abdominal pain, higher pancreatic morphological abnormalities, and were more prone to suffer from pancreatic exocrine and endocrine insufficiency as compared to un-mutated ICP cases [29]. Singh S et al. [30] reported that the N34S heterozygous mutation was relatively more prevalent as compared to the homozygous mutation, which was consistent with our study result. He also found that no significant phenotypical differences could be noticed between homozygote and heterozygote [30]. Threadgold J et al. suggested that the prevalence of the SPINK1 N34S mutation was higher in ICP cases (18%) as compared to normal individuals (2.5%), and familial ICP cases had a higher prevalence of this specific genetic mutation as compared to true or non-familial ICP patients (86% vs. 12.6%, respectively) too [26].

Limitations

We have some limitations in our study. We have neither assayed mutations in any other associated susceptible genes or ruled out any other susceptible locus mutations except targeted N34S mutation of the SPINK1 gene because of logistic issues and our study inclusion criteria. We have not stratified our cases into familial and true ICP cases, as our diagnosis of ICP was based only on the absence of known etiological factors, including heredity. We have assayed exocrine insufficiency only symptomatically and not objectively by subjecting the cases neither for fecal fat estimation or the elastase test due to the non-availability of these facilities in our region.

## Conclusions

From the study findings, we conclude that the SPINK1 N34S mutation was fairly common in ICP cases and the prevalence of the heterozygous mutation was relatively more common in comparison to the homozygous mutation. Although the disease onset was earlier in mutated cases as compared to un-mutated cases, and mutants had a relatively severe type of pancreatitis, clinical severity, and structural abnormality cannot be decided solely on the basis of this targeted genetic defect of the SPINK1 gene; rather, there might be some other hidden factors, a variable role of the environment, and other associated, unknown genetic mutations, which might have a contributory role in the phenotypical and morphological presentations of the disease, which needs to be meticulously addressed in future studies. However, a larger number of ICP cases should be subjected to detailed genetic testing to validate our findings in the future, and in the current context, we cannot advocate our study findings would be applicable globally, as we too observed variable types of results in our study population, which cannot be explained on the grounds of genetic mutation only and need further, long-term prospective studies to find out a definitive, conclusive answer in the future.

## References

[1] Steer ML, Waxman I, Freedman S (1995). Chronic pancreatitis. N Engl J Med.

[2] Somogyi L, Martin SP, Venkatesan T, Ulrich CD 2nd (2001). Recurrent acute pancreatitis: an algorithmic approach to identification and elimination of inciting factors. Gastroenterology.

[3] Etemad B, Whitcomb DC (2001). Chronic pancreatitis: diagnosis, classification, and new genetic developments. Gastroenterology.

[4] Yadav D, Lowenfels AB (2013). The epidemiology of pancreatitis and pancreatic cancer. Gastroenterology.

[5] Yadav D, Timmons L, Benson JT, Dierkhising RA, Chari ST (2011). Incidence, prevalence, and survival of chronic pancreatitis: a population-based study. Am J Gastroenterol.

[6] Pelaez-Luna M, Robles-Diaz G, Canizales-Quinteros S, Tusié-Luna MT (2014). PRSS1 and SPINK1 mutations in idiopathic chronic and recurrent acute pancreatitis. World J Gastroenterol.

[7] Gomez-Lira M, Bonamini D, Castellani C, Unis L, Cavallini G, Assael BM, Pignatti PF (2003). Mutations in the SPINK1 gene in idiopathic pancreatitis Italian patients. Eur J Hum Genet.

[8] Sobczyńska-Tomaszewska A, Bak D, Oralewska B (2006). Analysis of CFTR, SPINK1, PRSS1 and AAT mutations in children with acute or chronic pancreatitis. J Pediatr Gastroenterol Nutr.

[9] Whitcomb DC (2000). Genetic predispositions to acute and chronic pancreatitis. Med Clin North Am.

[10] Esposito I, Friess H, Büchler MW (2001). Molecular mechanisms in chronic pancreatitis [Article in German]. Zentralbl Chir.

[11] Shetty S, Leelakrishnan V, Krishnaveni S (2016). A study of SPINK 1 mutation and other clinical correlates in idiopathic chronic pancreatitis. JOP.

[12] Witt H, Luck W, Hennies HC (2000). Mutations in the gene encoding the serine protease inhibitor, Kazal type 1 are associated with chronic pancreatitis. Nat Genet.

[13] Audrézet MP, Chen JM, Le Maréchal C (2002). Determination of the relative contribution of three genes-the cystic fibrosis transmembrane conductance regulator gene, the cationic trypsinogen gene, and the pancreatic secretory trypsin inhibitor gene-to the etiology of idiopathic chronic pancreatitis. Eur J Hum Genet.

[14] Ammann RW (1997). A clinically based classification system for alcoholic chronic pancreatitis. Summary of an international workshop on chronic pancreatitis. Pancreas.

[15] Pfützer R, Myers E, Applebaum-Shapiro S (2002). Novel cationic trypsinogen (PRSS1) N29T and R122C mutations cause autosomal dominant hereditary pancreatitis. Gut.

[16] Mellitus D (2005). Diagnosis and classification of diabetes mellitus. Diabetes Care.

[17] Balakrishnan V, Unnikrishnan AG, Thomas V (2008). Chronic pancreatitis. A prospective nationwide study of 1,086 subjects from India. JOP.

[18] Balakrishnan V, Nair P, Radhakrishnan L, Narayanan VA (2006). Tropical pancreatitis - a distinct entity, or merely a type of chronic pancreatitis?. Indian J Gastroenterol.

[19] Garg PK, Tandon RK (2004). Survey on chronic pancreatitis in the Asia-Pacific region. J Gastroenterol Hepatol.

[20] Garg PK (2006). Chronic pancreatitis: the AIIMS, New Delhi experience. Chronic Pancreatitis and Pancreatic Diabetes in India.

[21] Midha S, Singh N, Sachdev V, Tandon RK, Joshi YK, Garg PK (2008). Cause and effect relationship of malnutrition with idiopathic chronic pancreatitis: prospective case-control study. J Gastroenterol Hepatol.

[22] Layer P, DiMagno EP (1999). Early and late onset in idiopathic and alcoholic chronic pancreatitis: different clinical courses. Surgical Clinics.

[23] Choudhuri G, Bhatia E, Sikora SS, Alexander G (2006). Tropical pancreatitis in North India. Chronic Pancreatitis and Pancreatic Diabetes in India.

[24] Chandak GR, Idris MM, Reddy DN, Mani KR, Bhaskar S, Rao GV, Singh L (2004). Absence of PRSS1 mutations and association of SPINK1 trypsin inhibitor mutations in hereditary and non-hereditary chronic pancreatitis. Gut.

[25] Chen JM, Mercier B, Audrezet MP, Raguenes O, Quere I, Ferec C (2001). Mutations of the pancreatic secretory trypsin inhibitor (PSTI) gene in idiopathic chronic pancreatitis. Gastroenterology.

[26] Threadgold J, Greenhalf W, Ellis I (2002). The N34S mutation of SPINK1 (PSTI) is associated with a familial pattern of idiopathic chronic pancreatitis but does not cause the disease. Gut.

[27] Drenth JP, te Morsche R, Jansen JB (2002). Mutations in serine protease inhibitor Kazal type 1 are strongly associated with chronic pancreatitis. Gut.

[28] Truninger K, Witt H, Köck J (2002). Mutations of the serine protease inhibitor, Kazal type 1 gene, in patients with idiopathic chronic pancreatitis. Am J Gastroenterol.

[29] Muller N, Sarantitis I, Rouanet M (2019). Natural history of SPINK1 germline mutation related-pancreatitis. EBioMedicine.

[30] Singh S, Choudhuri G, Agarwal S (2014). Frequency of CFTR, SPINK1, and cathepsin B gene mutation in North Indian population: connections between genetics and clinical data. Sci World J.

